# Assessment of expression of calcium signaling related lncRNAs in epilepsy

**DOI:** 10.1038/s41598-023-45341-7

**Published:** 2023-10-21

**Authors:** Mohammad Taheri, Ashkan Pourtavakoli, Solat Eslami, Soudeh Ghafouri-Fard, Arezou Sayad

**Affiliations:** 1 Institute of Human Genetics, Jena University Hospital, Jena, Germany; 2 Urology and Nephrology Research Center, Shahid Beheshti University of Medical Sciences, Tehran, Iran; 3 Genomic Research Center, Shahid Beheshti University of Medical Sciences, Tehran, Iran; 4 Dietary Supplements and Probiotic Research Center, Alborz University of Medical Sciences, Karaj, Iran; 5 Department of Medical Biotechnology, School of Medicine, Alborz University of Medical Sciences, Karaj, Iran; 6 Department of Medical Genetics, Shahid Beheshti University of Medical Sciences, Tehran, Iran

**Keywords:** Genetics, Neuroscience, Biomarkers

## Abstract

Calcium signaling is a metabolic pathway that is essential in neurons development and can be involved in the pathobiology of epilepsy. We assessed expression of three mRNA coding gene (*SLC1A1*, *SLC25A12*, and *ATP2B2*) and three related long non-coding RNAs (*LINC01231:1*, *lnc-SLC25A12-8:1* and lnc*-MTR-1:1*) from this pathway in 39 patients with refractory epilepsy and 71 healthy controls. Expression of all genes except for lnc-SLC25A12 was higher in total epileptic cases compared with controls (P values = 0.0002, < 0.0001, < 0.0001, 0.049 and 0.0005 for SLC1A1, SLC25A12, LINC01231, ATP2B2 and lnc-MTR-1, respectively. When we separately compared expression of genes among males and females, SLC1A1, SLC25A12, LINC01231 and lnc-MTR-1 showed up-regulation in male cases compared with male controls. Moreover, expressions of SLC1A1 and SLC25A12 were higher in female cases compared with female controls. Remarkably, SLC25A12 was found to have the highest sensitivity value (= 1) for differentiation of epileptic cases from controls. Moreover, lnc-MTR-1 and lnc-SLC25A12 were sensitive markers for such purpose (sensitivity values = 0.89 and 0.87, respectively). The highest value belonged to LINC01231 with the value of 0.76. Taken together, this study demonstrates dysregulation of calcium-signaling related genes in epileptic patients and suggests these genes as potential biomarkers for epilepsy.

## Introduction

Brain tissue is involved in many neurological disorders and one of these chronic disorders is epilepsy. Epilepsy is defined as a condition that patient has long-lasting generating seizures^1^. One third of patients with epilepsy do not respond to anti-seizure drugs and are known to have refractory epilepsy^2^. This condition affects quality of life and has adverse results in daily routines^3^.

Calcium signaling is a metabolic pathway that is essential in neurons development. Calcium acts as an important second messenger and contributes to the correct synapse activities. So, it is obvious that distraction in this signaling pathway may result in neurodevelopmental disorders^4^. Intrinsic burst firing, mediated by inward calcium ion is considered as the initiator of epileptic activity^5^. Calcium signaling is regulated by long non-coding RNAs (lncRNAs). Several calcium-binding proteins are subjected to regulation by these transcripts^6^.

Several of proteins and non-coding RNAs are involved in calcium signaling. For instance, *ATP2B2* gene codes a protein that removes bivalent calcium ions from cells and plays a critical role in calcium homeostasis. This gene is regulated by an lncRNA, namely *lnc-MTR-1*^7^. Solute Carrier Family 1 Member 1 (SLC1A1) is a glutamate transporter that is related with a number of signaling pathways including Transport of inorganic cations/anions. Mutations in this gene might be involved in the pathogenesis of schizophrenia^8^. Most notably, rare variants in this gene have been found to be associated with a kind of sensory epilepsy^9^.

*SLC25A12* codes for a mitochondrial aspartate/glutamate transporter which is principally expressed in neurons^10^. This protein is involved in the exchange of aspartate for glutamate and protons in the mitochondria^11^. Dysfunction of this protein leads to defects in the transport of aspartate into the cytoplasm, reduction of *N*-acetyl glutamate production in the cytoplasm and subsequent defects in the production of myelin^11^. SLC25A12 variants are involved in the pathogenesis of epilepsy^12,13^ possibly due to accumulation of glutamate which results in cellular injury^14^.

The role of calcium signaling related genes has been discussed in the context of brain disorders such as autism spectrum disorder^15^. Since calcium signaling has an efficient role in the neurodevelopmental disorders through regulation of normal neuronal activity, the related genes may play a role in the epilepsy as well. Thus, we analyzed expression of *SLC1A1*, *SLC25A12*, and *ATP2B2* genes that encode calcium signaling proteins that play a part in storing, regulation, and transmission of calcium in neurons. We also assessed expression of three lncRNAs that are related with these genes, namely *LINC01231:1*, *lnc-SLC25A12-8:1* and lnc*-MTR-1:1T*, respectively, as predicted by the LNCipedia^16^ and RNAcentral^17^ databases. The regulatory network constructed by lncRNA-mRNA interactions may affect epileptogenesis and can be considered as a putative target for therapeutic interventions, particularly in refractory epilepsy.

## Methods

### Patients and controls

In total, 40 patients with refractory seizures and 71 healthy persons (41 males and 30 females) were enrolled in the current study. Patients were classified according to the definition of International League Against Epilepsy (ILAE), “failure of adequate trials of two tolerated, properly selected and used antiepileptic drug plans (whether as monotherapy or in combination) to accomplish continued seizure freedom”^18^. Diagnosis was based on electroencephalogram and brain magnetic resonance imaging. Informed consent forms were signed by all participants. The study protocol was permitted by ethical committee of Shahid Beheshti University of Medical Sciences.

### Experiments

In this assay, we measured expression of six genes (coding and non-coding) which are shown in Table 1. For this purpose, we took blood samples from 39 refractory epileptic patients (5 cc of whole blood). Total RNA extraction and cDNA synthesis were accomplished by using RiboEx and SMOBIO kits, respectively. Then, real time PCR was performed in the ABI system using specific designed primers.

**Table 1:** Genes and primers.

Gene	RNA type	locus	F primer	R primer	Length of amplicon	Tm (°C)
*lnc-MTR-1:1*	lncRNA	chr1: 236,907,044–236,916,931	AGCCTGATGAACCAGTGTGCT	TCCAGCAATCTGCCTCTTTCCA	156	63
*ATP2B2*	Coding	3p25.3	GCGAGGGCAACGAAGGATGT	CCGTGACCAGGACCACACAGA	123	62
*SLC1A1*	Coding	9p24.2	CGGCGAGGAAAGGATGCGA	AGAGTTGAGAGGTTGCTGTGTTCT	130	63
*SLC25A12*	Coding	2q31.1	GCGGTCAAGGTGCAGACAACTA	AACGCTCTCCATCAACCTCAGTA	*94*	63
*LINC01231*	lncRNA	chr9: 3,181,589–3,200,500	TTCTGGAGGAAAGGGAAGAGATT	GGAGCCCAAGCACAGGTT	137	60
*lnc-SLC25A12-8:1*	lncRNA	chr2: 171,855,927–171,999,859	CAGGTGGGATGGAAGAAGCC	TACTGAGAATGAACTTGGGCAG	80	58

### Statistical analysis

Statistical analysis was accomplished in GraphPad Prism version 9.0 (GraphPad Software, La Jolla, CA, USA). Expression levels of three genes encoding ion channels and transporters, namely *SLC1A1*, *SLC25A12*, *ATP2B2* and their related lncRNAs, namely *LINC01231*, *lnc-SLC25A12*, and *lnc-MTR-1* were measured in the peripheral blood obtained from 39 patients with refractory epilepsy and 71 healthy controls. Expression levels were assessed using the comparative − delta Ct method. The normal/gaussian distribution of the values was evaluated using the Shapiro–Wilk test. Unpaired t test or non-parametric test (Mann–Whitney U test) was used to identify differentially expressed genes between two groups. Effects of main factors on gene expression were determined using the two-way ANOVA and Tukey post hoc tests. Correlations between expression levels of genes were measured using the Spearman’s rank correlation coefficient.

### Ethics approval and consent to participant

All procedures performed in studies involving human participants were in accordance with the ethical standards of the institutional and/or national research committee and with the 1964 Helsinki declaration and its later amendments or comparable ethical standards. Informed consent forms were obtained from all study participants. The study protocol was approved by the ethical committee of Shahid Beheshti University of Medical Sciences. All methods were performed in accordance with the relevant guidelines and regulations.

## Results

### General data

The study included 39 patients with refractory epilepsy (16 male subjects and 23 female subjects). Moreover, we included 71 healthy persons as controls (41 male subjects and 30 female subjects).

### Expression assays

**Figure 1:**
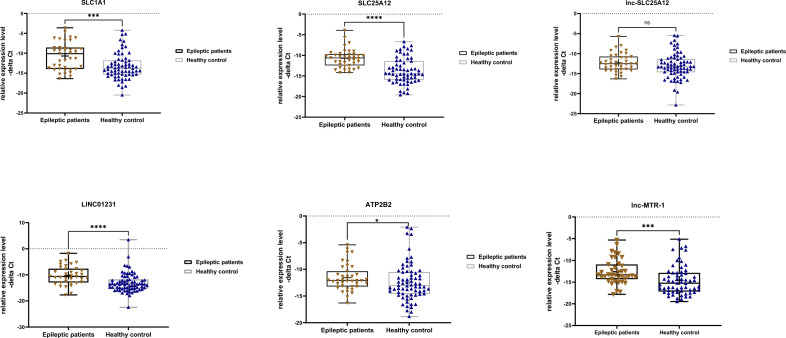
Expression of three genes encoding ion channels and transporters including SLC1A1, SLC25A12, ATP2B2 and their related long non-coding RNAs, namely LINC01231, lnc-SLC25A12, and lnc-MTR-1 in epilepsy patients and healthy controls as described by − delta Ct values. Following the normality test, unpaired t test (for SLC25A12 and lnc-SLC25A12) and non-parametric test (Mann–Whitney U test) (for SLC1A1, ATP2B2, LINC01231 and lnc-MTR-1) was used to identify differentially expressed genes between two groups (* < 0.05, ***P value < 0.001 and ****P value < 0.0001).

Expression levels of SLC1A1, SLC25A12, ATP2B2 and their related lncRNAs were significantly different between patients with epilepsy and healthy subjects (Fig. 1).

Group factor (disease) had a significant effect on expression levels of SLC1A1, SLC25A12, LINC01231 and lnc-MTR-1. On the other hand, gender factor and interaction between gender and group had no effect on expression levels of any of genes (Table 2); so, we did not perform post hoc tests for multiple comparisons.

**Table 2:** Graphpad prism output from analysis of effect of group and gender (tests of between-subjects effects) on expression of three genes encoding ion channels and transporters including SLC1A1, SLC25A12, ATP2B2 and their related long non-coding RNAs, namely LINC01231, lnc-SLC25A12, and lnc-MTR-1 in total epileptic patients compared to healthy controls.

Source of Variation	Group effect			Gender effect			Interactions		
	SS^1^	F^2^	P value	SS	F	P value	SS	F	P value
SLC1A1	164.7	14.59	**0.0002**	7.4	0.65	0.42	1.17	0.1	0.74
SLC25A12	221.2	27.97	** < 0.0001**	5.52	0.69	0.4	3.05	0.38	0.53
lnc-SLC25A12	16.35	1.96	0.16	9.33	1.12	0.29	1.79	0.21	0.64
LINC01231	205.4	15.7	**0.0001**	10.21	0.78	0.37	13.08	0.99	0.32
ATP2B2	20.92	2.02	0.15	0.13	0.01	0.9	6.95	0.67	0.41
lnc-MTR-1	111.3	11.29	**0.0011**	0.11	0.01	0.91	33.19	3.36	0.069

Two-way ANOVA and Tukey post hoc tests were used to analyze the effects of main factors (disease and gender) and their interaction on expression levels (*P value < 0.05, **P value < 0.01, ***P value < 0.001 and ****P value < 0.0001).

^1^Sum of Squares.

^2^F of Variance.

Significant values are in bold.

Expression of all genes except for lnc-SLC25A12 was higher in total epileptic cases compared with controls (P values = 0.0002, < 0.0001, < 0.0001, 0.049 and 0.0005 for SLC1A1, SLC25A12, LINC01231, ATP2B2 and lnc-MTR-1, respectively (Table 3). When we separately compared expression of genes among males and females, SLC1A1, SLC25A12, LINC01231 and lnc-MTR-1 showed up-regulation in male cases compared with male controls. Moreover, expressions of SLC1A1 and SLC25A12 were higher in female cases compared with female controls.

**Table 3:** Expression ratio (fold change) of three genes encoding ion channels and transporters and their related long non-coding RNAs, namely LINC01231, lnc-SLC25A12, and lnc-MTR-1 in epileptic patients compared to healthy controls.

Genes	Parameters	Total patients vs. Controls (50 vs. 50)	Male patients vs. Male Controls (16 vs. 30)	Female patients vs. Female Controls (23 vs. 41)	Female patients vs. Male patients (23 vs. 16)	Female controls vs. Male Controls (41 vs. 16)
*SLC1A1*	Expression ratio (95% CI)	12.9 (2.37–17.6)	7.19 (1.06–48.16)	5.28 (1.06–26.17)	0.58 (0.08–4.19)	0.79 (0.17–3.55)
	Adjusted P value	**0.0002**	**0.039**	**0.037**	0.89	0.97
*SLC25A12*	Expression ratio (95% CI)	8 (3.63–17.5)	10.8 (2.2–52.7)	6.5 (1.63–25.99)	0.55 (0.1–2.9)	0.91 (0.25–3.34)
	Adjusted P value	** < 0.0001**	**0.0009**	**0.0033**	0.79	0.99
*lnc-SLC25A12*	Expression ratio (95% CI)	1.81 (0.82–4.02)	1.46 (0.28–7.41)	2.13 (0.55–8.28)	0.78 (0.14–4.28)	0.53 (0.15–1.9)
	Adjusted P value	0.13	0.92	0.46	0.98	0.58
*LINC01231*	Expression ratio (95% CI)	8.1 (3.38–20.82)	13.19 (1.65–103.9)	4.66 (0.8–26.9)	0.94 (0.1–8.5)	2.66 (0.55–12.8)
	Adjusted P value	** < 0.0001**	**0.008**	0.1	0.99	0.36
*ATP2B2*	Expression ratio (95% CI)	2.07 (1–4.19)	2.75 (0.45–16.79)	1.31 (0.28–5.93)	0.65 (0.09–4.31)	1.37 (0.33–5.61)
	Adjusted P value	**0.049**	0.46	0.96	0.93	0.93
*lnc-MTR-1*	Expression ratio (95% CI)	4.78 (2.05–10.48)	10.5 (1.71–64)	1.99 (0.43–9.18)	0.45 (0.07–2.9)	2.4 (0.55–10.48)
	Adjusted P value	**0.0005**	**0.005**	0.63	0.68	0.4

Significant values are in bold.

Since most of Ct values were not normally distributed, we used non parametric Spearman’s correlations between RNA expression levels among the epileptic patients and healthy controls. When analyzing the correlation between expression levels of genes, we noticed that the most robust correlations were between lnc-MTR-1/ATP2B2, ATP2B2/lnc-SLC25A12 and lnc-MTR-1/lnc-SLC25A12 pairs among epileptic cases with correlation coefficients of 0.73, 0.73 and 0.68, respectively (Table 4).

**Table 4:** Spearman’s correlations between RNA expression levels among the epileptic patients (*N* = 39) and healthy controls (*N* = 71).

	SLC25A12		lnc-SLC25A12		LINC01231		ATP2B2		lnc-MTR-1	
	Patients	Controls	Patients	Controls	Patients	Controls	Patients	Controls	Patients	Controls
SLC1A1	0.41**	0.6**	0.65**	0.57**	0.27*	0.59**	0.6**	0.55**	0.56**	0.43**
SLC25A12			0.58**	0.26*	0.48**	0.57**	0.6**	0.4**	0.47**	0.57**
lnc-SLC25A12					0.62**	0.45**	0.73**	0.6**	0.68**	0.23*
LINC01231							0.45**	0.65**	0.32*	0.52**
ATP2B2									0.73**	0.45**

*P < 0.05.

**P < 0.001.

**Figure 2:**
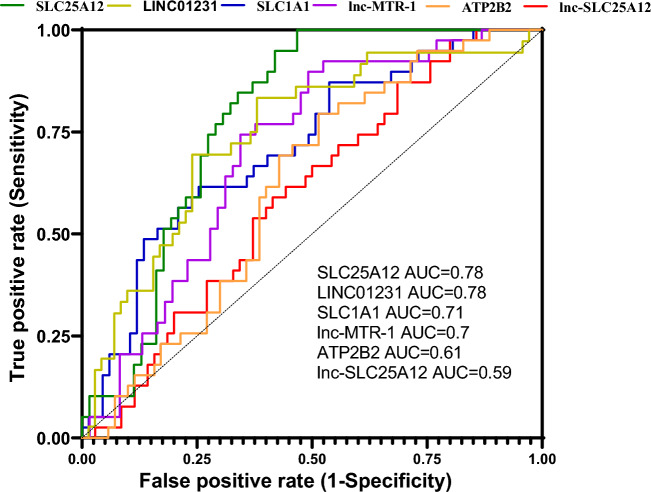
ROC curves of six studied genes in patients with epilepsy disease.

AUC values for mentioned genes ranged from 0.78 for SLC1A1 and LINC01231 to 0.59 for lnc-SLC25A12 (Fig. 2).

Remarkably, SLC25A12 was found to have the highest sensitivity value (= 1) for differentiation of epileptic cases from controls. Moreover, lnc-MTR-1 and lnc-SLC25A12 were sensitive markers for such purpose (sensitivity values = 0.89 and 0.87, respectively). The highest value belonged to LINC01231 with the value of 0.76 (Table 5).

**Table 5:** ROC curve analysis for transcript levels of six studied genes in patients with epilepsy.

SLC25A12				LINC01231				SLC1A1				lnc-MTR-1				lnc-SLC25A12				ATP2B2			
AUC ± SEM	Sensitivity	Specificity	P value	AUC ± SEM	Sensitivity	Specificity	P value	AUC ± SEM	Sensitivity	Specificity	P value	AUC ± SEM	Sensitivity	Specificity	P value	AUC ± SEM	Sensitivity	Specificity	P value	AUC ± SEM	Sensitivity	Specificity	P value
0.78 ± 0.04	1	0.53	< 0.0001	0.74 ± 0.05	0.69	0.76	< 0.0001	0.71 ± 0.05	0.61	0.74	0.0003	0.7 ± 0.05	0.89	0.5	0.0006	0.59 ± 0.05	0.87	0.31	0.1	0.61 ± 0.05	0.79	0.48	0.049

## Discussion

Calcium signaling has an important role in the neurological disorders. In fact, the triad of excitotoxicity, calcium and mitochondria has been found to be involved in synaptic neurodegeneration^19^. Malfunction of proteins related with this pathway is followed by mitochondrial calcium toxicity and excitotoxic dendritic loss and hence neurodegeneration^19^. Meanwhile, neurons channel proteins and synaptic plasticity are two targets of lncRNAs which are intertwined with epilepsy pathogenesis^20^.

It is also confirmed that astrocytes have a key role in controlling the conformation of the extracellular fluids, and can directly cooperate with neurons by discharging gliotransmitters. Notably, astrocytic intracellular Calcium signals increase discharge of signaling elements, either via synaptic or non-synaptic routes. Thus, astrocytic calcium signals have crucial roles in epileptogenesis^21^. Calbindin-D28K, a member of calcium binding protein family has been found to be down-regulated in the cortical tubers of patients with tuberous sclerosis complex associated with refractory epilepsy^22^. However, expression of other calcium-related proteins has not been assessed in refractory versus non-refractory epilepsy.

In the current study, we measured expression of a number of mRNA coding genes and lncRNAs in epileptic patients. Expression of all genes except for lnc-SLC25A12 was higher in total epileptic cases compared with controls. When we separately compared expression of genes among males and females, SLC1A1, SLC25A12, LINC01231 and lnc-MTR-1 showed up-regulation in male cases compared with male controls. Moreover, expressions of SLC1A1 and SLC25A12 were higher in female cases compared with female controls. A previous study has reported significant elevation of SLC1A1 expression in dentate granule cells from rats with spontaneous seizure compared with similar cells from control rats^23^. Moreover, expression of these transcripts have been shown to be high in human dentate granule cells from patients with temporal lobe epilepsy and as well as in dysplastic neurons in cortical dysplasia compared with non-dysplastic neurons of control tissues obtained from autopsy^23^. Thus, the results of our study provide further evidence for involvement of SLC1A1 in the pathobiology of epilepsy. We also detected up-regulation of *LINC01231*, the lncRNA that interacts with the *SLC1A1* mRNA. This lncRNA has also been shown to be upregulated in major depressive disorder cases and down-regulated in Alzheimer’s disease patients^24^. Thus, it is involved in a variety of neurologic disorders.

In line with our findings, mutations in *SLC25A12* gene are associated with infantile epileptic encephalopathy, psychomotor retardation, hypomyelination of the CNS and seizures^25^. Moreover, two polymorphisms within *SLC25A12* gene (rs2292813 and rs2056202) are associated with autism spectrum disorder^26^. In spite of up-regulation of this gene in epileptic cases, expression of the related lncRNA with this gene (lnc-SLC25A12) was not different between cases and controls. This finding might suggest that the role of this mRNA coding gene in epilepsy is independent from the related lncRNA.

We also noticed that the most robust correlations were between lnc-MTR-1/ATP2B2, ATP2B2/lnc-SLC25A12 and lnc-MTR-1/lnc-SLC25A12 pairs among epileptic cases. In fact, the strongest correlation was detected between ATP2B2 and its related lncRNA, i.e. lnc-MTR-1. However, the correlations between other mRNA coding genes and their related lncRNAs were not so strong.

Remarkably, SLC25A12, lnc-MTR-1 and lnc-SLC25A12 were found to be sensitive markers for differentiation of epileptic cases from controls. However, the specificity of these transcripts were not appropriate. Taken together, this study demonstrates dysregulation of calcium-signaling related genes in epileptic patients and suggests these genes as potential biomarkers for epilepsy.

### Supplementary Information

Supplementary Information.

## Data Availability

All data generated or analysed during this study are included in this published article (and its Supplementary Information files).
